# Regulatory T Cells Resist Cyclosporine-Induced Cell Death via CD44-Mediated Signaling Pathways

**DOI:** 10.1155/2015/614297

**Published:** 2015-09-10

**Authors:** Shannon M. Ruppert, Ben A. Falk, S. Alice Long, Paul L. Bollyky

**Affiliations:** ^1^Division of Infectious Diseases and Geographic Medicine, Department of Medicine, 300 Pasteur Drive, Stanford University School of Medicine, Stanford, CA 94305-5107, USA; ^2^Benaroya Research Institute, 1201 Ninth Avenue, Seattle, WA 98101, USA

## Abstract

Cyclosporine A (CSA) is an immunosuppressive agent that specifically targets T cells and also increases the percentage of pro-tolerogenic CD4+Foxp3+ regulatory T cells (Treg) through unknown mechanisms. We previously reported that CD44, a receptor for the extracellular matrix glycosaminoglycan hyaluronan (HA), promotes Treg stability in IL-2-low environments. Here, we asked whether CD44 signaling also promotes Treg resistance to CSA. We found that CD44 cross-linking promoted Foxp3 expression and Treg viability in the setting of CSA treatment. This effect was IL-2 independent but could be suppressed using sc-355979, an inhibitor of Stat5-phosphorylation. Moreover, we found that inhibition of HA synthesis impairs Treg homeostasis but that this effect could be overcome with exogenous IL-2 or CD44-cross-linking. Together, these data support a model whereby CD44 cross-linking by HA promotes IL-2-independent Foxp3 expression and Treg survival in the face of CSA.

## 1. Introduction

In healthy individuals, immunologic tolerance is maintained by populations of regulatory cells, including Foxp3+ regulatory T cells (Treg). Treg are a subset of T cells that suppress autoreactive effector T cells (Teff). The absence or depletion of Treg leads to multisystemic autoimmunity in mice and humans [1]. In addition, adoptive transfer of Treg can rescue the healthy phenotype [2, 3].

Treg rely on Interleukin 2 (IL-2) for their suppressive function [4]. IL-2 promotes Foxp3 expression and Treg suppressor functions via signal transducer and activator of transcription 5 (Stat5) signaling [5–10]. However, Foxp3 suppresses autocrine IL-2 production [6], such that Treg rely on other cell types, particularly activated Teff cells, as a source of IL-2. Along with effects on Treg, IL-2 activates Teff lymphocytes as well and promotes their proliferation.

In addition to IL-2, other factors are also known to support Treg maintenance* in vivo* and Treg are present in IL-2^−/−^ as well as CD25^−/−^ mice, though both strains do develop autoimmunity [11]. Furthermore, ambient IL-2 levels in circulation and in peripheral tissues [12–14] are often a fraction of what Treg require in culture. Finally, while Treg in culture are anergic,* in vivo* their proliferation rate is high [15, 16]. Together, these data suggest that additional factors exist that support Treg maintenance* in vivo.*

Cyclosporine A (CSA) is an immunosuppressive agent that inhibits T cell proliferation by suppressing IL-2 synthesis [17]. CSA inhibits calcineurin-dependent IL-2 production by forming a complex with cyclophilin that inhibits calcineurin phosphatase induced upon T cell activation [18]. In turn, the transcription factor nuclear factor of activated T cells (NFAT) remains in a phosphorylated state and, subsequently, cannot upregulate IL-2 gene expression [19].

The effectiveness of CSA treatment may reflect, in part, changes in the ratio of Treg to Teff cells [20]. Previous clinical and* in vivo* studies have found that treatment with CSA can promote Foxp3 expression in Treg [21, 22].* In vivo* human data likewise suggests that CSA treatment results in higher levels of circulating Treg than in healthy donors [23]. These data suggest that Treg may have a survival advantage in certain contexts over Teff cells in the face of CSA. However, the mechanisms that underlie the relative sparing of Treg in the setting of CSA treatment are unclear.

One tissue factor known to promote Treg homeostasis in low-IL-2 environments is hyaluronan (HA), an extracellular matrix (ECM) glycosaminoglycan. We and others have demonstrated that high molecular weight HA (HMW-HA) (>1 × 10^6^ kDa), characteristic of healing, and uninjured tissues [24] promote the function and persistence of Treg [25–27]. These effects are dependent on HA polymer length and cross-linking of the primary HA receptor, CD44 [28], the expression of which is elevated on Treg [25]. In light of these data, we have proposed that HMW-HA cross-links CD44 and thereby provides Treg with homeostatic signals in injured and healing tissues [26].

We previously reported that CD44 cross-linking allows Treg to resist CSA-mediated cell death [28]. However, the underlying mechanisms remained unclear, as both Foxp3 and CSA are well known to efficiently suppress IL-2 production [6, 18].

Here, we have evaluated the hypothesis that CD44 cross-linking bypasses CSA treatment by recapitulating aspects of IL-2R signaling in the absence of IL-2. We found that CD44 cross-linking promotes Foxp3 expression in an IL-2-independent but Stat5-dependent manner. Moreover, we report that inhibition of HA synthesis impairs Treg homeostasis but that this effect could be overcome with exogenous IL-2 or CD44-cross-linking. Together, these data support a model whereby CD44 cross-linking by HA promotes IL-2-independent and Stat5-dependent Foxp3 expression and Treg survival in the face of CSA.

## 2. Materials and Methods 

### 2.1. Mice

Foxp3-GFP C57BL/6 mice were the kind gift of Dr. Alexander Rudensky. CD25 deficient C57BL/6 (CD25^−/−^) mice were purchased from The Jackson Laboratory (Bar Harbor, ME). Foxp3-GFP mice were crossed with CD25^−/−^ mice to generate Foxp3-GFP.CD25^−/−^ mice at our institution. All mice were maintained in specific pathogen-free AAALAC-accredited animal facilities at the BRI and Stanford University and handled in accordance with institutional guidelines.

### 2.2. Isolation of Leukocyte Populations

Mouse leukocyte populations were isolated from inguinal, axial, and brachial lymph nodes and spleen cells from 6- to 8- week-old mice. CD4+ T cell populations were isolated using a CD4+ Isolation Kit (Miltenyi Biotec) as per the manufacturer's instructions. Foxp3/GFP+ and Foxp3/GFP-T-cells were then isolated using either a FACS-Vantage Flow Cytometer Cell Sorter or BD FACS Aria. Purity of the resulting cell fractions was reliably >99.9% Foxp3/GFP+.

### 2.3. Reagents and Cell Lines

Hyaluronan of 1.5 × 10^6^ kDa molecular weight (HMW-HA) was provided by Genzyme. Cyclosporine A (CSA) was obtained from Sigma-Aldrich (St. Louis, MO). Recombinant mouse IL-2R alpha antibody (R&D Systems), IL-2 (Chiron), and neutralizing antibody against CD25 (3C7, BioLegend) were used. HMW-HA conjugated to BSA was used for plate-bound HMW-HA activation studies, as described previously [29].

### 2.4. Flow Cytometry Analysis

CD44 cross-linking of Treg was performed as follows: 1 × 10^6^ cells/well were cultured in a 96-well round-bottom tissue culture plate. Cells were then treated under different stimulation conditions as follows: media alone, IL-2 (100 IU/mL), and/or plate-bound anti-mouse CD44 Ab (10 *μ*g/mL, IM7, BD). Alternatively, for analysis following HMW-HA treatment, 1 × 10^6^ cells/well in 96-well round-bottom tissue culture plates were serum starved for 2.5 hours before the assay. The cells were then resuspended in RPMI 1640 (Invitrogen) supplemented with 10% FBS (Hyclone, Logan, UT), 100 *μ*g/mL Penicillin, 100 U/mL Streptomycin, 50 *μ*M *β*me, 2 mM glutamine, and 1mM sodium pyruvate (Invitrogen) prior to transfer to plates previously coated with 50 *μ*g/mL BSA-conjugated HMW-HA. On the third day, cells were harvested and washed prior to analysis. Where indicated, the following reagents were incubated at 37°C in a CO_2_ incubator with the cells 30 minutes prior to cross-linking CD44: CSA (50 ng/mL or at concentrations indicated) and neutralizing Ab against CD25 (100 *μ*g/mL; R&D Systems, Cat number AB-223-NA).

Flow cytometry experiments used the following fluorochrome-labeled antibodies: CD3e (145-2C11), CD4 (RM4-5), CD25 (PC61.5), and CD44 (IM7) from BD-Biosciences and eBioscience. Labeled cells resuspended in FACS buffer were analyzed on a LSRII flow cytometer. Analysis was performed using CELLQuest (BD) and FlowJo (Treestar Inc., Ashland, OR) software. Of note, all FACS plots and MFI values shown were gated on live cells unless otherwise noted.

### 2.5. Murine Treg Activation Assays

96-well flat bottom tissue culture plates were precoated with anti-CD3 Ab (0.5 *μ*g/mL) and anti-CD44 Ab (1 *μ*g/mL) where relevant. Soluble anti-CD28 Ab was used at 0.5 *μ*g/mL. Coating with HMW-HA was treated as a second step. Plates were washed with PBS and then either 100 *μ*L of 100 *μ*g/mL HMW-HA or 100 *μ*L of 10% BSA in PBS was added and the plates were incubated at 37°C for 2 hours. Plates were again washed with PBS prior to the addition of 150,000 CD4+CD25+ Treg in RPMI-10 complete medium. Where noted, CSA (50 ng/mL) or soluble IL-2R*α* (5 *μ*g/mL) was added at the inception of the experiment. No exogenous IL-2 was added unless otherwise noted. After three days Treg were stained and analyzed by flow cytometry.

### 2.6. Statistical Analysis

Graphs were prepared using JMP software (SAS Institute, Cary, NC) and GraphPad Prism (La Jolla, CA). Significance was assessed using paired* t* tests or ANOVA unless otherwise noted.

## 3. Results

### 3.1. CSA Increases Treg Percentages in a CD44 Dependent Manner

To evaluate CSA effects on Treg viability and Foxp3 expression, we used tissues isolated from transgenic mice expressing GFP in concert with Foxp3. We purified CD4+ T cells from these animals and activated them in culture for 72 hours. Our goal was to test whether CSA increased the fraction of Foxp3+ Treg among total CD4+ cells* in vitro*, as has been reported* in vivo* [21, 22].

**Figure 1:**
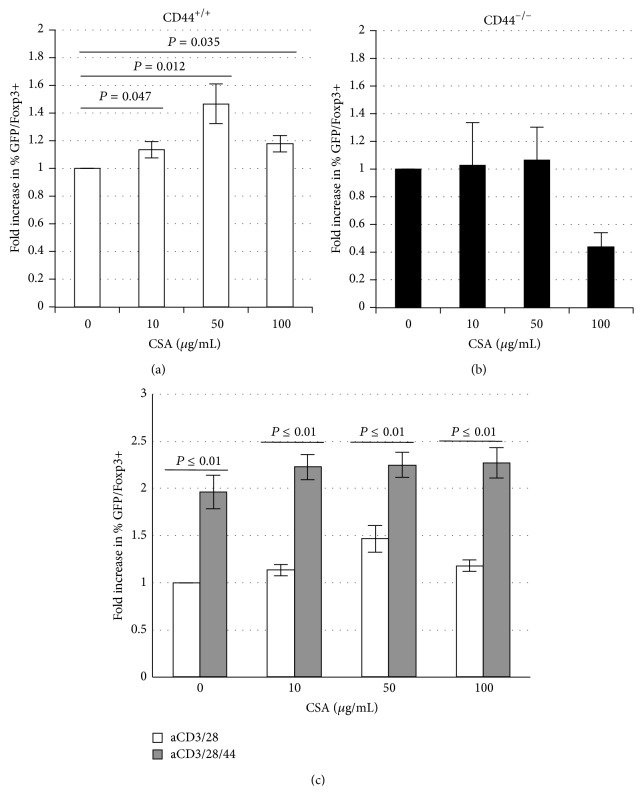
*CSA increases Treg percentages in a CD44 dependent manner.* Fold increase (FI) in the percentage of GFP/Foxp3+ cells of (a) CD44-containing or (b) CD44-deficient, murine CD4+ T cells after 3 days of culture in the settings of anti-CD3 and anti-CD28 with or without CSA in various concentrations. (c) Fold increase (FI) in percentage of GFP/Foxp3+ cells after 3 days of culture in the setting of anti-CD3 and anti-CD28 alone or in conjunction with anti-CD44 Ab. *n* = 5 replicate wells.

We observed that CSA treatment significantly increased the percentage of Foxp3+ Treg among total CD4+ T cells. This effect was significant at a range of CSA concentrations but was most pronounced at moderate CSA levels (50 *μ*g/mL) (Figure 1(a)). This effect is due to increased cell death among the GFP/Foxp3−, conventional T cell fraction and not the GFP/Foxp3+ Treg fraction (data not shown).

No such increase in the GFP/Foxp3 fraction was seen in cells isolated from CD44^−/−^ mice (Figure 1(b)). We also observed that CD44-cross-linking heightened the percentage of Foxp3+ Treg, even at high concentrations of CSA (Figure 1(c)). These data indicate that (1) Treg are relatively resistant to CSA, compared to Teff cells, (2) that this resistance is CD44 dependent, and (3) can be magnified by CD44 cross-linking.

### 3.2. Both CD44 Cross-Linking and IL-2 Promote Foxp3 Expression and Treg Persistence Despite CSA Treatment

The effects of CD44 cross-linking on Foxp3 levels were analogous to what one might expect to see with IL-2 supplementation. We therefore directly compared the effects of CD44 cross-linking and IL-2 supplementation on Treg cultured in the setting of CSA. To this end, we freshly isolated CD4+GFP/Foxp3+ Treg from GFP/Foxp3 transgenic mice and activated these for three days in the absence or presence of CSA.

**Figure 2:**
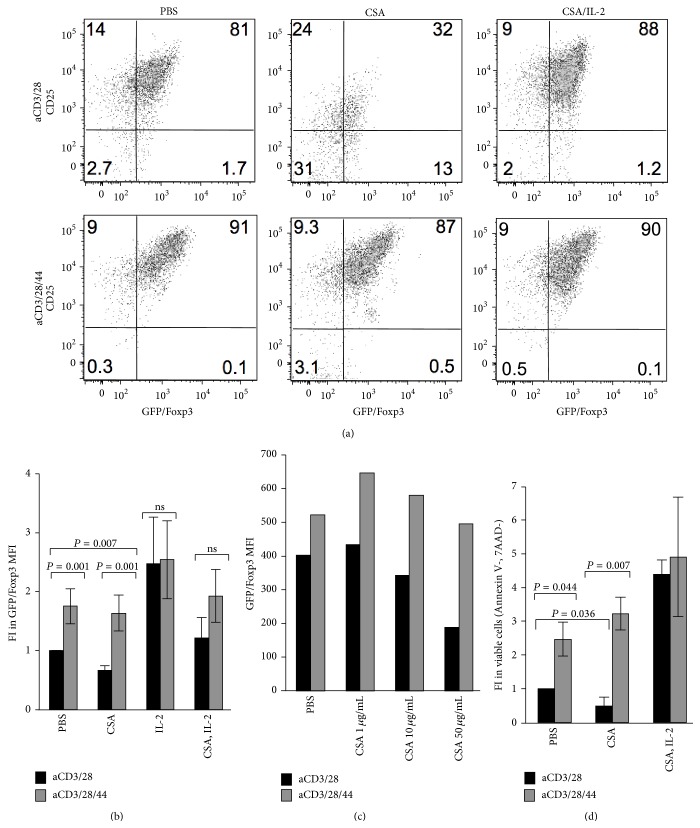
*CD44 cross-linking and IL-2 both promote Foxp3 expression and Treg persistence despite CSA treatment.* (a) Representative flow cytometric analysis of CD25 labeled and GFP/Foxp3+ cells after 3 days in culture in the presence of anti-CD3 and anti-CD28 alone or with the addition of anti-CD44, and with or without CSA (50 ng/mL) alone or together with IL-2 (20 IU/mL). (b) Fold increase (FI) in GFP/Foxp3 MFI after 3 days of culture in the presence of anti-CD3 and anti-CD28 alone or in conjunction with anti-CD44 Ab, with or without CSA (50 ng/mL) alone or together with IL-2 (20 IU/mL). *N* = 4 independent experiments, among these are included Figure 2(a). (c) Fold Increase in GFP/FoxP3 MFI in the presence of anti-CD3 and anti-CD28 alone, or in conjunction with anti-CD44 and increasing concentrations of CSA. Data are representative of two experiments. (d) Fold increase in the fraction of viable GFP/FoxP3+ cells (Annexin V-, 7AAD-) upon culture with aCD3/28 or aCD3/28/44 with or without CSA (50 ng/mL) alone or together with IL-2 (20 IU/mL). *N* = 4 experiments among these are included in Figure 2(a) and the other experiments are in Figure 2(b).

We observed that Foxp3 and CD25 expression was abrogated by treatment with CSA but that both CD44 cross-linking and supplementation with 100 IU/mL of IL-2 maintained Foxp3 expression. Indeed, CD44 cross-linking and IL-2 supplementation were roughly equivalent in their ability to promote Treg homeostasis in the setting of CSA (Figures 2(a) and 2(b)). Of note, this beneficial effect of CD44 cross-linking on Foxp3 levels was predicated on the concomitant presence of TCR signals; CD44 cross-linking in the absence of aCD3/28 did not promote resistance to CSA (data not shown).

As with our observation using total CD4+ T cells in Figure 1, CD44 cross-linking on purified Treg likewise supported maintenance of Foxp3 expression across a range of CSA concentrations (Figure 2(c)). In addition, we saw comparable effects when we assessed Treg viability upon CSA treatment, as measured by 7-AAD and Annexin V staining. However, CSA treatment of Treg in the absence of CD44 cross-linking led to a significant decrease in viability (Figure 2(d)).

Together, these data indicate that CD44 cross-linking and IL-2 supplementation exert parallel effects on Treg, allowing them to escape cell death caused by treatment with CSA.

### 3.3. CD44 Cross-linking Promotes Foxp3 Expression in an IL-2-Independent Manner

We previously reported that CD44 cross-linking by HMW-HA promoted Treg maintenance in the absence of exogenous IL-2 [26, 28]. As part of those studies, we observed that CD44 cross-linking allowed Treg to resist CSA-mediated cell death [28]. We had proposed that CD44 cross-linking promoted increased IL-2 production by Treg.

However, this interpretation of the data was problematic for several reasons. First, Foxp3 is known to efficiently suppress IL-2 production [6]. While we did detect IL-2 in Treg cultures [28], this may be better explained by the propensity of some GFP/Foxp3+ Treg to revert to being GFP/Foxp3− Teff cells capable of producing IL-2 [30]. Indeed, upon intracellular staining of these cells for IL-2, we observed that the IL-2 producing cells were uniformly GFP/Foxp3− (data not shown). Second, CSA also suppresses IL-2 production by both Treg and Teff alike in an efficient manner [18]. Consistent with this, the level of IL-2 we detected in these cultures by ELISA, in the 5–20 pg/mL range, would be insufficient to support Treg homeostasis. We therefore sought to better ascertain whether CD44-mediated support of Treg homeostasis was indeed IL-2 independent.

**Figure 3:**
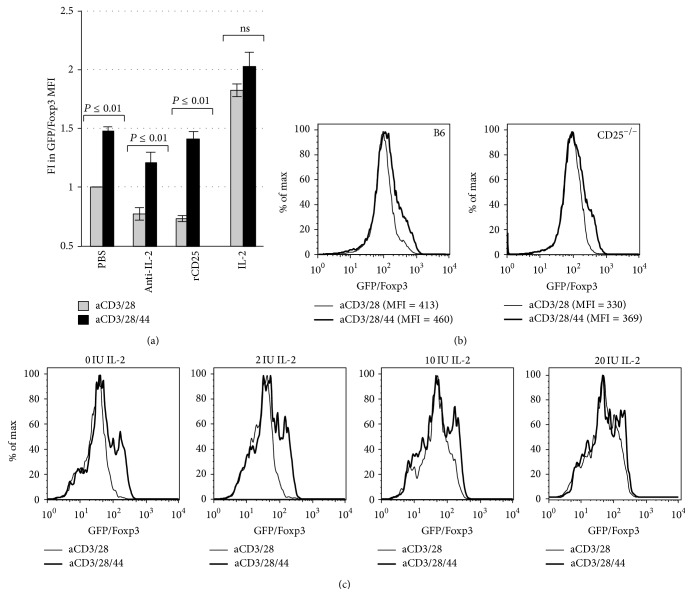
*CD44 cross-linking promotes Foxp3 expression in an IL-2-independent manner*. (a) Fold Increase (FI) in GFP/FoxP3 MFI for Treg activated with anti-CD3 and anti-CD28 Ab alone or in conjunction with plate-bound anti-CD44 Ab with or without anti-IL-2 Ab (Anti-IL-2), recombinant CD25 (rCD25), or IL-2 (*n* = 7). (b) Representative histograms demonstrating GFP/Foxp3 expression by Treg isolated from GFP/Foxp3 knock-in mice on a conventional B6 background mice or on a CD25^−/−^ background (B6 GFP/Foxp3.CD25^−/−^ mice) following 3 days of culture with anti-CD3 and anti-28 alone or in conjunction with plate-bound anti-CD44. (c) Representative histograms illustrating GFP/FoxP3 expression of Treg following 3 days of culture with anti-CD3 and anti-CD28 alone, or in conjunction with plate-bound CD44 Ab, and with or without varying doses of IL-2. Data are representative of two experiments.

We first neutralized any IL-2 that might be produced in our Treg activation cultures by adding recombinant CD25 (rCD25), the high affinity IL-2 receptor, or antibodies directed at IL-2. However, these reagents did not completely negate the beneficial effect of CD44 cross-linking on Foxp3 expression (Figure 3(a)).

We then obtained CD4+GFP/Foxp3+ Treg from transgenic GFP/Foxp3 mice lacking CD25, the high affinity IL-2 receptor (GFP/FoxP3.CD25^−/−^ mice). We found that the absence of CD25 had minimal impact on CD44-mediated Foxp3 expression, as the wild type B6 mice and CD25^−/−^ Treg cells had almost equal Foxp3 expression levels (Figure 3(b)).

Because IL-2 effects on Treg homeostasis are dose-dependent, we next tested whether CD44 cross-linking effects were additive with IL-2 supplementation from 0–20 IU/mL (0–4000 pg/mL). We found that the effects of CD44 cross-linking on Foxp3 persistence were most pronounced at low levels of IL-2 and that CD44 cross-linking was functionally equivalent to adding high levels of IL-2 (10–20 IU/mL, equivalent to 2000–4000 pg/mL) to these cultures (Figure 3(c)).

Together, these data support the conclusion that CD44 cross-linking potentiates Foxp3 expression of Treg in an IL-2 and CD25 independent manner.

### 3.4. Inhibition of HA Synthesis Impairs Treg Homeostasis Which Can Be Overcome with Exogenous IL-2 or CD44-Cross-Linking

We next determined whether the effect of CD44 cross-linking on Foxp3 expression could be induced by its natural ligand HA and whether this source of HA was paracrine or autocrine in origin.

**Figure 4:**
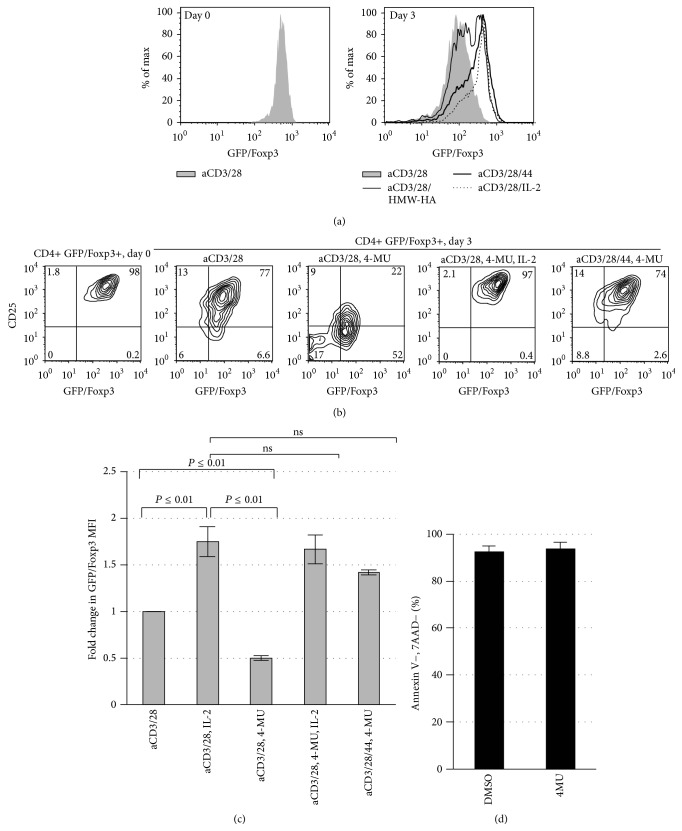
*Inhibition of HA synthesis impairs Treg homeostasis which can be overcome with exogenous IL-2 or CD44-cross-linking*. (a) Representative histograms of GFP/FoxP3 expression of Treg following 3 days of culture in the presence of anti-CD3 and anti-CD28 alone or with IL-2, CD44 cross-linking, or exogenous plate-bound HA. *N* = 3 independent experiments. (b) Representative FACS plots illustrating GFP/Foxp3 and CD25 expression on Day 0 immediately following isolation of CD4+GFP/Foxp3+ Treg from murine splenocytes and following 3 days of culture with anti-CD3 and anti-CD28 Ab alone or in conjunction with plate-bound anti-CD44 Ab, the HA synthesis inhibitor 4-MU, and/or IL-2. (c) Fold change in GFP/Foxp3 MFI for the same conditions as in (b), here for *N* = 3 independent experiments. (d) Viability (the percentage of GFP/Foxp3+ cells negative for 7AAD and Annexin V) for Treg cultured in the setting of either DMSO or 4MU.

To test this, we incubated CD4+/Foxp3+ Treg for 72 hours in the presence of HMW-HA, plate-bound CD44 antibody, or IL-2, and examined Foxp3 expression. We observed that cross-linking CD44 receptor through either plate-bound CD44 antibody or HMW-HA promoted Foxp3 expression in CD4+ Tregs in a manner similar to IL-2 (Figure 4(a)).

To evaluate whether the HA that promoted Foxp3 expression was paracrine or autocrine in nature, we abrogated endogenously produced HA in CD4+ Treg by culturing them in the presence of 4-methylumbelliferone (4-MU), a specific hyaluronan synthase inhibitor [31]. Activated Treg cultured with 4-MU were unable to maintain Foxp3 expression over 72 hours (Figure 4(b)). As expected, IL-2 supplementation could completely rescue Foxp3 expression. Moreover, when these cells were cultured with anti-CD44 antibody, Foxp3 expression was maintained at levels similar to controls. These observations were confirmed in multiple experimental replicates (Figure 4(c)). Taken together, these results demonstrate that both endogenous and exogenous HA can potentiate Foxp3 expression and that the loss of HA production can be compensated for by cross-linking of the HA receptor, CD44.

### 3.5. CD44-Mediated Foxp3 Expression Is Stat5-Dependent

Many of the cytokines known to support Treg homeostasis, including IL-2, signal through Stat5. We therefore evaluated whether the promotion of Foxp3 persistence by CD44 cross-linking is mediated by Stat5 signaling.

**Figure 5:**
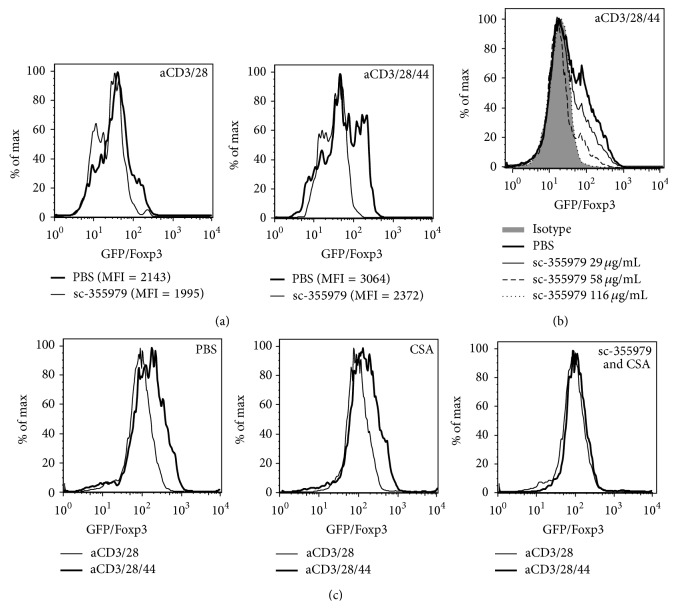
*CD44-mediated Foxp3 expression is Stat5-dependent*. (a) Representative histograms for GFP/Foxp3 expression of CD4+GFP/Foxp3+ Treg following culture in the presence of sc-35597, a selective pStat5 inhibitor. (b) Representative histograms for GFP/Foxp3 following 3 days of culture with aCD3/28/44 together with increasing concentrations of sc-35597. (c) Representative histograms depicting GFP/Foxp3 persistence following culture with CSA (50 ng/mL) alone or in conjunction with sc-35597. Data for (a–c) are representative of at least 3 experiments.

We observed that the ability of CD44 cross-linking to promote Foxp3 expression was lost upon treatment with sc-355979, a selective inhibitor of pStat5 (Figure 5(a)). This capacity of sc-355979 to overcome CD44-mediated Foxp3 expression was dose dependent (Figure 5(b)). Finally, whereas CSA treatment alone did not affect Foxp3 expression induced by CD44, combined treatment with CSA and pStat5 inhibition did impair CD44-mediated Foxp3 expression (Figure 5(c)).

Taken together, these results indicate that the CD44-mediated promotion of Foxp3 expression and the ability of CD44 cross-linking to bypass CSA treatment depend on Stat5 activation.

## 4. Discussion

Cyclosporine (CSA) is a widely used immunosuppressant that selectively targets T cells, thereby preventing antigen-specific immune processes like transplant rejection [32, 33]. There are data that suggest that CSA may selectively spare Treg and it may be that this contributes to CSA efficacy [20–22]. Here, we have identified a novel, CD44-mediated and IL-2 independent mechanism for how Treg may escape CSA suppression.

A role for CD44 in Treg persistence in the face of CSA would be consistent with other lines of evidence supporting a role for CD44 in homeostasis. We previously published that CD44 and HMW-HA promote Treg homeostasis in low IL-2 environments [25–27] and it was recently reported that complexes of CD44 and Galectin-9 promote the stability and function of Treg in a SMAD2/3 signaling dependent manner [34]. CD44 also contributes to Treg function [25, 27, 29] and the capacity to bind HMW-HA is known to characterize the most potent subset of Treg [25]. Of note, CD44 cross-linking did not rescue GFP/Foxp3−, conventional T cells from CSA effects in our hands. We speculate that this may reflect the fact that these cells are typically CD44lo. Also of note, it is highly unlikely that expansion of Treg is responsible for this effect given that it is generally accepted that Treg do not expand* in vitro* in the absence of high dose IL-2 and potent antigenic signals. Induction of Treg from conventional T cells is also highly unlikely given the absence of high dose IL-2, potent antigenic signals, and TGF*β* [6].

The constitutive expression of CD44 may also promote homeostasis of additional CD44^hi^ T cell subsets. For example, CD44 has been implicated in the homeostasis of memory T cells, which are also CD44^hi^ [35]. In addition to total CD44 levels, the distribution of CD44 on the cell surface, and the make-up of CD44 variant isoforms could also impact T cell homeostasis.

These data may be relevant to understanding seemingly contradictory data indicating that CSA does not uniformly spare Treg (reviewed in [36]). Specifically, there are some reports that CSA inhibits Foxp3 mRNA expression [37] and induces the reversion of Treg to pro-inflammatory phenotypes [38]. Similarly,* in vivo* studies in which mice underwent MHC-mismatch bone marrow transplantation demonstrated that CSA treatment impaired expansion of Treg and reduced overall Foxp3 expression [39]. More recently, it was shown that CSA treatment preferentially inhibits antigen-specific Treg [40]. Miroux and colleagues found that CSA decreased both the activity and proliferation of Treg [41]. Our data suggest that the dose of CSA may impact the ability of Treg to resist CSA. Consistent with this, Kawai and colleagues demonstrated that* in vivo* administration of CSA inhibits the proliferation of Treg at high doses, but not so at low doses [42]. Besides this, our data also support roles for antigenic signals, co-stimulation though CD44, and therefore the local inflammatory milieu as factors that influence Treg susceptibility to suppression by CSA.

Our data support a role for CD44-mediated, IL-2 independent, promotion of Treg resistance to CSA and implicate Stat5 signaling in this effect [4, 43]. Stat5 signaling is known to be essential for both the maintenance of Foxp3 expression and the suppressive function of Treg* in vivo* [44, 45]. This is underscored by the observation that Stat5 knockout mice exhibit a loss of Treg [46]. Additionally, overexpression of Stat5 in IL-2 knockout mice can restore* in vivo* Treg numbers [46]. However, avenues for Stat5 signaling not directly involving IL-2 are likely to be important for Treg homeostasis. Our current efforts are therefore focused on determining whether CD44 cross-linking directly phosphorylates Stat5 or, alternatively, whether these effects are indirectly mediated.

## 5. Conclusions

We have identified a role for CD44, a receptor for HA, in promoting Treg resistance to CSA. This effect is IL-2 independent but can be suppressed by inhibition of Stat5 phosphorylation. Moreover, we find that inhibition of HA synthesis impairs Treg homeostasis but that this effect can be overcome with exogenous IL-2 or CD44-cross-linking. Together, these data support a model whereby CD44 cross-linking by HA promotes IL-2-independent Foxp3 expression and Treg survival in the face of CSA.

## Abbreviations

ECM: Extracellular matrix
HA: Hyaluronan
HMW-HA: High-molecular-weight hyaluronan
Treg: Foxp3+ regulatory T cell.
